# Luminescence Properties of Self-Aggregating Tb^III^-DOTA-Functionalized Calix[4]arenes

**DOI:** 10.3389/fchem.2018.00001

**Published:** 2018-01-30

**Authors:** Florian Mayer, Sriram Tiruvadi Krishnan, Daniel T. Schühle, Svetlana V. Eliseeva, Stéphane Petoud, Éva Tóth, Kristina Djanashvili

**Affiliations:** ^1^Department of Biotechnology, Delft University of Technology, Delft, Netherlands; ^2^Centre de Biophysique Moléculaire, UPR 4301 Centre National de la Recherche Scientifique, Université d'Orléans, Orléans, France; ^3^Le Studium, Loire Valley Institute for Advanced Studies, Orléans, France

**Keywords:** calix[4]arenes, DOTA-chelates, lanthanides, terbium, luminescence, optical imaging

## Abstract

Self-aggregating calix[4]arenes carrying four DOTA ligands on the *upper rim* for stable complexation of paramagnetic Gd^III^-ions have already been proposed as MRI probes. In this work, we investigate the luminescence properties of Tb^III^-DOTA-calix[4]arene-4OPr containing four propyl-groups and compare them with those of the analog substituted with a phthalimide chromophore (Tb^III^-DOTA-calix[4]arene-3OPr-OPhth). We show that, given its four aromatic rings, the calix[4]arene core acts as an effective sensitizer of Tb-centered luminescence. Substituents on the *lower rim* can modulate the aggregation behavior, which in turn determines the luminescence properties of the compounds. In solid state, the quantum yield of the phthalimide derivative is almost three times as high as that of the propyl-functionalized analog demonstrating a beneficial role of the chromophore on Tb-luminescence. In solution, however, the effect of the phthalimide group vanishes, which we attribute to the large distance between the chromophore and the lanthanide, situated on the opposite rims of the calix[4]arene. Both quantum yields and luminescence lifetimes show clear concentration dependence in solution, related to the strong impact of aggregation on the luminescence behavior. We also evidence the variability in the values of the critical micelle concentration depending on the experimental technique. Such luminescent calix[4]arene platforms accommodating stable lanthanide complexes can be considered valuable building blocks for the design of dual MR/optical imaging probes.

## Introduction

Calix[4]arenes were initially proposed as artificial enzyme mimics in the late 70s (Gutsche and Muthukrishnan, 1978) and today represent versatile building blocks with potential for application in industrial, technical and biomedical fields, ranging from wastewater treatment (Konczyk et al., 2016) to medical imaging (Schühle et al., 2011; Sreenivasu Mummidivarapu et al., 2015). The multifunctional constitution of calix[4]arenes consists of four phenol moieties forming a cup-shaped structure with an *upper* and a *lower rim*. Functionalization of the rims can be done in accordance to the desired properties, including solubility, amphiphilicity, and metal complexation characteristics. The calixarene core is a synthetic backbone that ideally can play an active role in various applications. One example is represented by artificial ion-channels, where the hydrophobic channel-like cavity is of high importance for ion translocation through the membrane (Lawal et al., 2009). Enzyme mimics are also thought to benefit from this basket that resembles hydrophobic pockets in enzymes and serves for positioning of the substrate (Blanchard et al., 1998; Baldini et al., 2012). Despite those exciting examples, most often calix[4]arenes are simply used as platforms to attach the groups of interest in a predetermined spatial arrangement (Modi et al., 2016), which downgrades the core to a mere steric support without further function. Especially, calix[4]arene derivatives intended for medical applications often lack active participation of the core structure itself.

**Figure 1:**
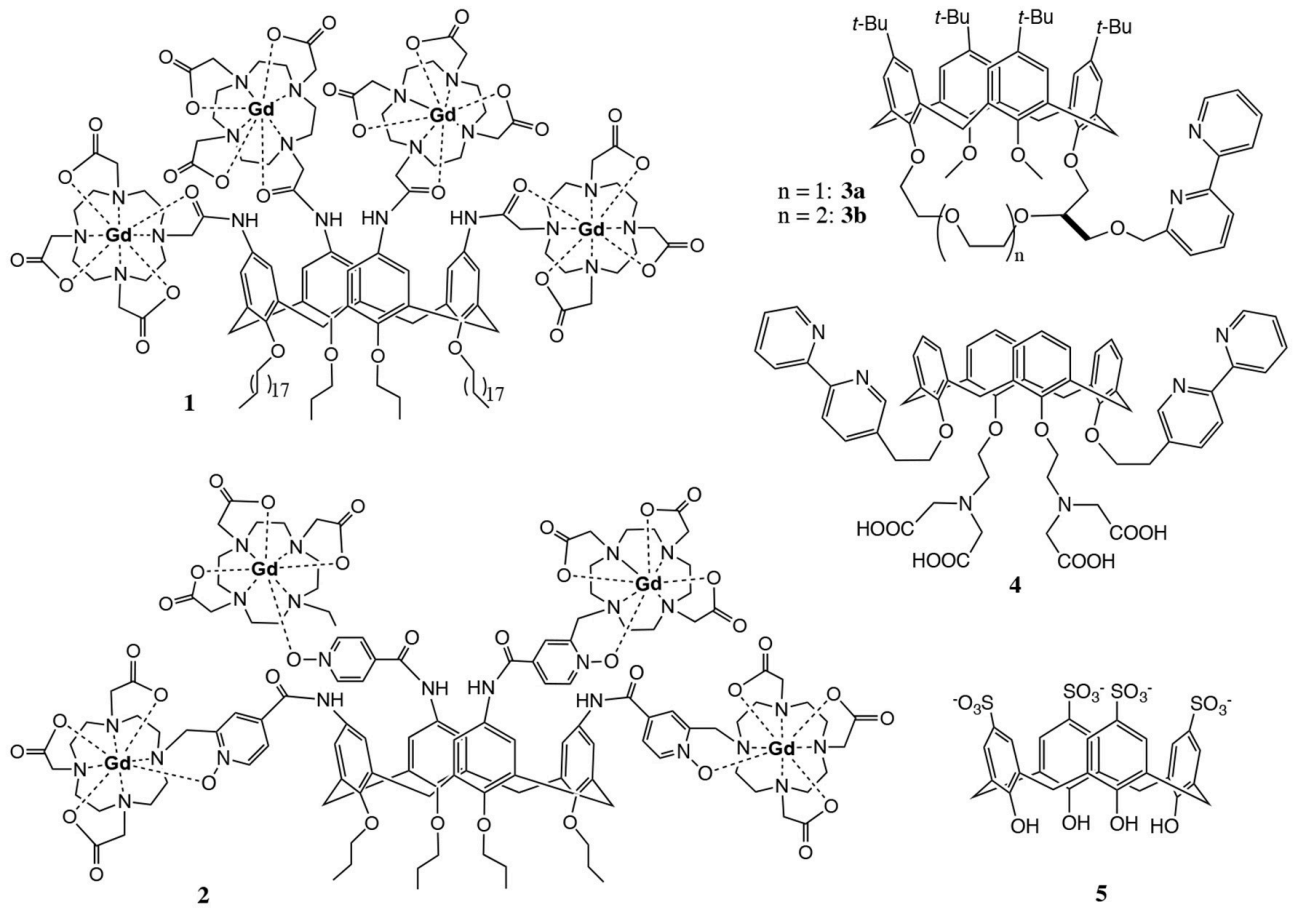
Examples of calix[4]arenes designed for imaging applications.

We have been interested in designing imaging agents based on calix[4]arenes for a long time. In the context of contrast agent development for magnetic resonance imaging (MRI), the *upper rim* of the core structure was decorated with four DOTA (1,4,7,10-tetraazacyclododecane-1,4,7,10-tetraacetate) chelating ligands to provide stable complexation of paramagnetic Gd^III^ ions. The Ln^III^ complexes of these calix[4]arenes **1** and **2** (Figure 1) are amphiphilic molecules with a very polar *upper rim* and an apolar *lower rim*, tuneable depending on the alkylation of the phenolic OH-groups.

This amphiphilic nature confers them high water solubility due to micelle formation in polar solvents and also opens the possibility for labeling lipid bilayers. Complexes **1** (Schühle et al., 2009) and **2** (Schühle et al., 2010) exhibit high longitudinal proton relaxivities *r*_1_ (expressed in s^−1^mM^−1^ of Gd^III^), especially when they form micelles, interact with human serum albumin or are incorporated in lipid bilayers.

The four chelating units of calix[4]arenes **1** and **2** can also be complexed with luminescent Ln^III^ ions to design optical probes. Eventually, the combination of Gd^III^ and luminescent Ln^III^ complexes within the same molecular platform could lead to dual MRI/optical imaging probes. To use calix[4]arenes as optical imaging agents, the sensitisation of the Ln^III^ ions has to be ensured. Usually, this is done by surrounding Ln^III^ ion with appropriate aromatic chromophoric units that are able to efficiently absorb excitation energy and transfer it to the lanthanide ion (Bünzli, 2015). An alternative strategy is to exploit the intermolecular energy transfer from an antenna incorporated in e.g., a micellar interior to the Ln^III^ ion (Ln = Tb or Eu) (Escabi-Perez et al., 1977; Darwent et al., 1993).

A few literature examples indicate the potential of calix[4]arene derived compounds for optical imaging in combination with lanthanides (Bunzli et al., 1993, 1998). Fischer et al. designed calix[4]arenes **3** and **4** (Figure 1) functionalized with bipyridyl moieties in the *lower rim* which participate in the complexation of Ln^III^ ions (Ln^III^ = Eu^III^, Tb^III^) and are at the same time responsible for the excitation of the luminescent center (Fischer et al., 2000). This architecture limits the number of lanthanide-binding sites to one, thus eliminating the great advantage of calix[4]arenes to deliver several active centers per molecule. In addition, the poor water solubility of these apolar compounds hampers thorough investigation of the luminescence properties and strongly limits biological applicability. In another example, calix[4]arene **5** containing *p*-sulfonate groups was found to exhibit fluorescence upon complexation with Tb^III^ at pH > 10.8 (Sato et al., 1993). The unexpected optical properties of this simple water soluble calix[4]arene were explained by a sandwich structure. The Tb^III^ ion is between two complexing molecules which provide eight coordinating oxygens and push away potential hydration water molecules. However, none of these systems ensure sufficiently stable metal complexation appropriate for biological use.

**Figure 2:**
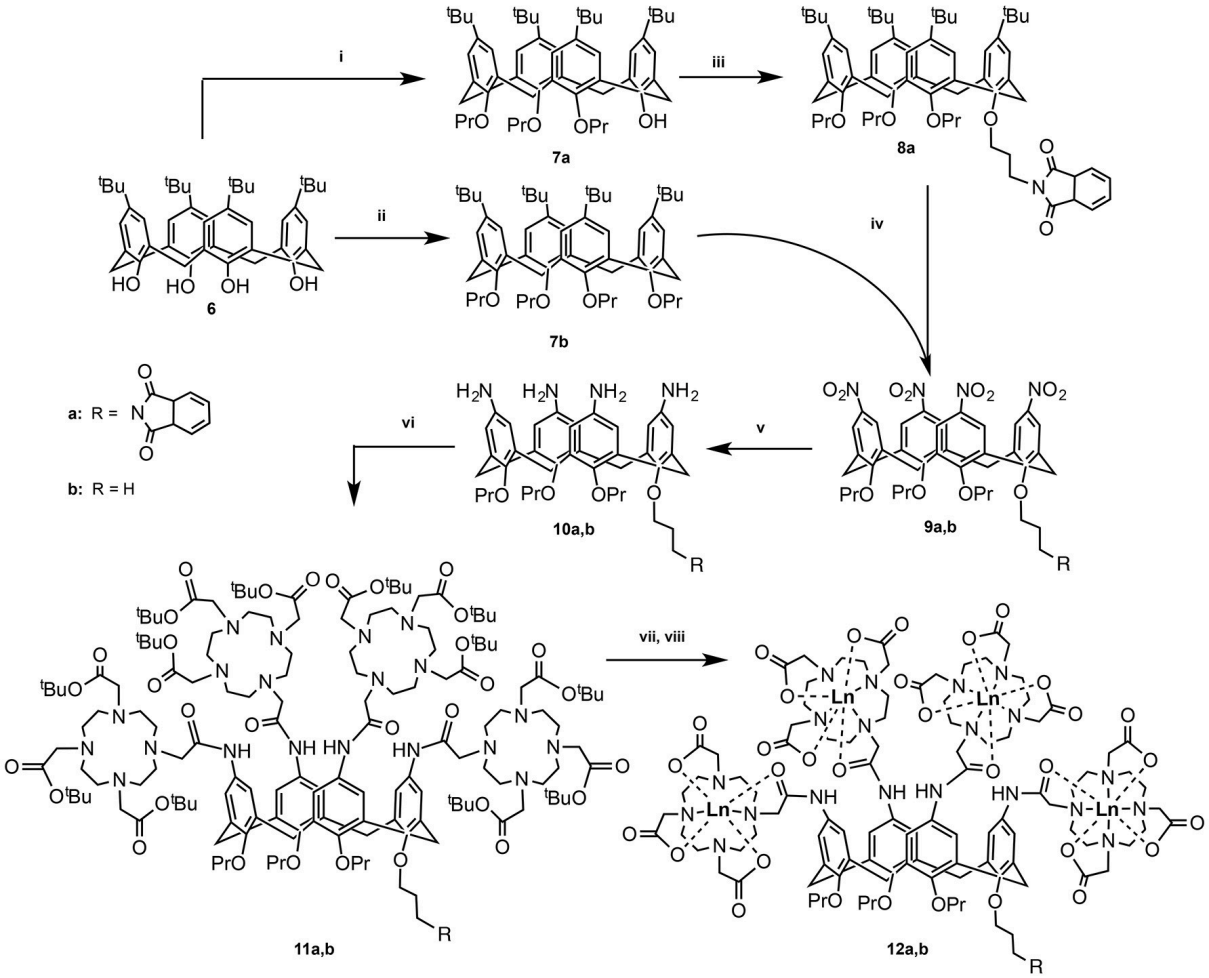
Synthetic pathway for compounds **12a** and **12b**: (i) DMF, Ba(OH)_2_, BaO, propylbromide, RT, 1 day; (ii) DMF, NaH, propylbromide, RT, 5 days (Gutsche and Lin, 1986); (iii) DMF, NaH, N-(3-bromo)propylphthalimide, RT, 5 days (Lalor et al., 2007); (iv) (a) CH_2_Cl_2_, AcOH, HNO_3_, 0°C, 4 h (Kelderman et al., 1992); (v) MeOH, hydrazine, Raney-Ni, reflux, 6.5 h (Klimentová and Vojtíšek, 2007); (vi) CH_3_CN, tris-*t*ert-butoxycarbonylmethyl-10-carboxymethyl-1,4,7,10-tetraazacyclodocecane (tris-^*t*^Bu-DOTA), Hünig's base, hydroxybenzotriazole, EDC, RT, 36 h; (vii) CH_2_Cl_2_, TFA; (viii) TbCl_3_, H_2_O, pH 5.5.

In the objective of adapting our DOTA-derivative calix[4]arene platform designed for MRI purposes (Schühle et al., 2010) to lanthanide luminescence, we have functionalized one site of the *lower rim* with a phthalimide chromophore while the three other sites bear propyl-groups (DOTA-calix-3OPr-OPhth, Figure 2, **11a**). Keeping in mind the aggregation tendency of the functionalized calix[4]arenes and the literature examples showing that a direct coordination of the antenna to the luminescent center is not always an absolute requirement (Bonnet et al., 2010), we hypothesized that *lower rim* conjugation with a phthalimide moiety could be sufficient to sensitize Tb-luminescence. The aggregation and the photophysical properties of the Tb^III^ complex have been studied in comparison to the Tb^III^-DOTA-calix-4OPr analog (Figure 2, **11b**) with four propyl residues at the *lower rim*. We show that aggregation has a strong impact on the luminescence behavior. In addition, our data provide a piece of evidence of the variability in the values of the critical micelle concentration depending on the experimental technique.

## Materials and methods

### General

All reagents and anhydrous solvents used during the synthesis were of commercial quality. 5,11,17,23-Tetra-(*t*-butyl)-25-hydroxy-26,27,28-tripropoxy-calix[4]arene (**6**) was prepared according to the literature (Gutsche and Iqbal, 1990). The *lower rim* of the calix[4]arene backbone was modified in accordance to the described procedures for the propylation (**7a,b)** of the hydroxylic groups (Gutsche and Lin, 1986), as well as condensation of one of the hydroxyls (**8a**) with N-(3-bromo)propylphthalimide (Lalor et al., 2007). The steps, preceding the final conjugation with the DOTA-units (Schühle et al., 2009) included nitration (**9a,b**) of the *upper rim* of the calix[4]arene backbone (Kelderman et al., 1992) followed by the reduction (**10a,b**) of the nitro groups to the amines (Klimentová and Vojtíšek, 2007). ^1^H NMR spectra were recorded at 25°C on Bruker Avance-400 spectrometer operating at 400.13 MHz and analyzed using Bruker™ TopSpin 2.1 software. The chemical shifts are reported in δ (ppm) using tetramethylsilane (TMS) as an internal reference. Ultra-filtration was performed with a Millipore stirred cell using an Amicon cellulose acetate membrane. All HPLC measurements were carried out on a Shimadzu LC-20 system consisting out of an LC-20AT pump, Sil-20A HT autosampler, CTO-20AC column oven, SPD-M20A PDA detector, CBM-20A controller, and a Waters Fraction Collector III; data processing was carried out using Shimadzu Lab Solutions. Both analytical and preparative methods were carried out operating at 40°C using eluents A: H_2_O (95%), AcCN (5%), TFA (0.1%) and B: H_2_O (5%), AcCN (95%), TFA (0.1%). Mobile phase gradient started with 75% A and 25% B, after 18 min followed by a change linear to 58% A and 42% B, after 2 min a change linear to 100% B, which was hold for 0.5 min and then chanced back to starting conditions stabilized for 3.5 min. Analytical measurements used a Waters Xterra 4.6 × 150 mm column and an injection volume of 1 μL, flow was 1 mL/min. Preparative HPLC was performed using Xbridge™ PrepShield RP18-OBD C18-19 × 150 mm column. Mass spectrometry analysis was done with electron spray ionization technique on Waters Qtof Premier MS using a NE-1000 syringe pump for direct infusion; data processing was carried out using Waters Masslynx. Qualitative luminescence measurements were done on a Jasco J815 CD spectrometer using 100 μL of sample in a 3 × 3 mm quartz cuvette. UV absorption spectra were measured on a UV2401 PC Shimadzu spectrometer. For quantitative luminescence measurements, the samples (either powders or solutions in Milli-Q water at concentration 2, 0.2, and 0.04 mM) were placed into 2.4 mm quartz capillaries and measured on a Horiba-Jobin-Yvon Fluorolog 3 spectrofluorimeter equipped with visible (220–800 nm, photon-counting unit R928P) and NIR (950–1,450 nm, photon-counting units H10330-45 from Hamamatsu or DSS-IGA020L Jobin-Yvon solid-state InGaAs detector, cooled to 77 K) detectors. All spectra were corrected for the instrumental functions. Luminescence lifetimes of Tb^III^-complexes were determined under excitation at 300 nm provided by a Xenon flash lamp monitoring the signal at 545 nm (^5^D_4_→^7^F_5_ transition). Quantum yields were measured according to an absolute method using an integration sphere (GMP SA). Each sample was measured several times under slightly different experimental conditions. Estimated experimental error for quantum yields determination is 10%. Nile red (NR) fluorescence measurements were performed on a Jasco J-815 CD spectrometer. The temperature was controlled using a Jasco PFD 4252/15 Peltier temperature unit. All samples contained Nile red in 2 μM concentrations and were excited at 550 nm. The maximum Nile red emission wavelength (λ_max_) was determined as a function of the calix[4]arene concentration.

### Synthesis

**General Procedure for the Amide Coupling of the Amino-Calix[4]arenes (10a,b) to tris-1,4,7-*tert*-Butoxycarbonylmethyl-10-Carboxymethyl-1,4,7,10-Tetraazacyclododecane (tris-*t*-Bu-DOTA):** To a suspension of EDC (2.2 mmol), HOBt (2.2 mmol), and tris-*t*-butyl-DOTA (2.2 mmol) in 20 mL DMF 1.1 mL dry DIPEA was added under N_2_ atmosphere and the resulting mixture was stirred for 20 min. A solution of the corresponding calix[4]arene (170 μmol) in 5 mL DMF was added and the reaction mixture was stirred for 2 days before removing the solvent *in vacuo*. The remaining solid was dissolved in CH_2_Cl_2_ (50 mL), washed 2× with brine, 1× with 0.1 N NaOH, and then with water until the pH of the aqueous phase was neutral. The organic phase was dried over Na_2_SO_4_ and the solvent was evaporated. The crude products **11a** and **11b** were purified from EtOH by ultrafiltration over a 1 kDa membrane with the yield of 80% (410 mg) and 69% (336 mg), respectively.

*5,11,17,23-Tetrakis(tris-4,7,10-t-butoxycarbonylmethyl-1,4,7,10-tetraazacyclododec-1-yl-acetamidyl)-25,26,27-tripropoxy-28-phthalimidopropoxycalix[4]arene (**11a**)*: ^1^H-NMR (300 MHz, DMSO-d_6_, 100°C): δ = 0.98 (12 H, *t, J* = 5.2 Hz, CH_3_), 1.40, 1.46 (108 H, 2 s, t-Bu), 1.88 (8 H, sext, *J* = 5.2 Hz, CH_2_CH_3_), 2.74–3.30 (100 H, N-CH_2_CO, N-CH_2_-CH_2_-N, Ar-CH_2_-Ar), 3.87 (8H, *t, J* = 5.2 Hz, O-CH_2_), 4.43 (4 H, d, *J* = 9.9 Hz, Ar-CH_2_-Ar), 6.86 (8 H, brs, Ar-H), 9.27 (4 H, brs, NH). ESI-HRMS: calc.: *m/z* = 755.4765 (M+4H)^4+^, found: 755.4870.

*5,11,17,23-Tetrakis(tris-4,7,10-tert-butoxycarbonylmethyl-1,4,7,10-tetraazacyclododec-1-yl-acetamidyl)-25,26,27,28-tetrapropoxycalix[4]arene (**11b**):* ^1^H-NMR (300 MHz, DMSO-d_6_, 100°C): δ = 0.98 (12 H, *t, J* = 5.2 Hz, CH_3_), 1.42, 1.46 (108 H, 2 s, t-Bu), 1.88 (8 H, sext, *J* = 5.2 Hz, CH_2_CH_3_), 2.74–3.30 (100 H, N-CH_2_CO, N-CH_2_-CH_2_-N, Ar-CH_2_-Ar), 3.87 (8H, *t, J* = 5.2 Hz, O-CH_2_), 4.43 (4 H, d, *J* = 9.9 Hz, Ar-CH_2_-Ar), 6.86 (8 H, br s, Ar-H), 9.27 (4 H, brs, NH). ESI-HRMS: calc.: *m/z* = 718.7185 (M+4H)^4+^, found: 718.7282.

**General Procedure for the Deprotection of the *t*-Bu-DOTA-calix[4]arenes:** The *t*-Bu protected calix[4]arene (150 μmol) was dissolved in CH_2_Cl_2_ (10 mL) and TFA (10 mL) was added slowly. The reaction mixture was stirred at ambient temperature overnight and subsequently the solvents were removed in vacuum. The obtained solid was re-dissolved in 1 mL of water and freeze-dried to obtain the product as a yellow fluffy powder. The yield was not determined due to the fact that there were still TFA salts in the product, which did not interfere with the next step of the synthesis and could be removed during the purification step of the final product (see below).

*5,11,17,23-Tetrakis(tris-4,7,10-carboxymethyl-1,4,7,10-tetraazacyclododec-1-yl-acetamidyl)-25,26,27-tripropoxy-28-phthalimidopropoxycalix[4]arene:* ^1^H-NMR (300 MHz, DMSO-d_6_, 100°C): δ = 0.89 (12 H, *t, J* = 6.9 Hz, CH_3_), 1.80 (8 H, sext, *J* = 6.9 Hz, CH_2_CH_3_), 3.01–3.61 (100 H, N-CH_2_CO, N-CH_2_-CH_2_-N, Ar-CH_2_-Ar), 3.76 (8H, *t, J* = 6.9 Hz, O-CH_2_), 4.32 (4 H, d, *J* = 12.3 Hz, Ar-CH_2_-Ar), 6.91 (8 H, brs, Ar-H), 9.53 (4 H, brs, NH).

*5,11,17,23-Tetrakis(tris-4,7,10-carboxymethyl-1,4,7,10-tetraazacyclododec-1-yl-acetamidyl)-25,26,27,28-tetrapropoxycalix[4]arene:* ^1^H-NMR (300 MHz, DMSO-d_6_, 100°C): δ = 0.89 (12 H, *t, J* = 6.9 Hz, CH_3_), 1.80 (8 H, sext, *J* = 6.9 Hz, CH_2_CH_3_), 3.01–3.61 (100 H, N-CH_2_CO, N-CH_2_-CH_2_-N, Ar-CH_2_-Ar), 3.76 (8H, *t, J* = 6.9 Hz, O-CH_2_), 4.32 (4 H, d, *J* = 12.3 Hz, Ar-CH_2_-Ar), 6.91 (8 H, brs, Ar-H), 9.53 (4 H, brs, NH).

**General Procedure for the Complexation of Tb**^III^**-ions in the DOTA-Functionalized Calix[4]arenes (12a,b):** The ligands (150 μmol) obtained after the deprotection of *t-*Bu groups were dissolved in water (5 mL) and the pH was adjusted to 5.5 by the addition of 1M NaOH. Then the TbCl_3_ (660 μmol) was added as an aqueous solution and the pH was kept constant using Metrohm Dosimeter 665. After the consumption of NaOH stopped, the solution was stirred overnight and then freeze-dried to result in the crude product that was purified by prep-HPLC. Due to the paramagnetic nature of the Tb^III^ ions, no NMR investigations of the products were possible, therefore MS spectra were taken and compared to the predicted isotopic patterns of the compounds, which were found in a good agreement with the experimental values (Supplementary Figures 1, 2).

*Tb*^*III*^*-complex of 5,11,17,23-tetrakis(tris-4,7,10-carboxymethyl-1,4,7,10-tetraazacyclododec-1-yl-acetamidyl)-25,26,27-tripropoxy-28-phthalimidopropoxycalix[4]arene (Tb-**12a**):* ESI-HRMS: calc.: *m/z* = 989.9197 (M+2H)^3+^, found: 989.8913.

*Tb*^*III*^*-complex of 5,11,17,23-tetrakis(tris-4,7,10-carboxymethyl-1,4,7,10-tetraazacyclododec-1-yl-acetamidyl)-25,26,27,28-tetrapropoxycalix[4]arene (Tb-**12b**):* ESI-HRMS: calc.: *m/z* = 941.2423 (M+3H)^3+^, found: 941.3101.

## Results and discussion

The synthetic routes of both compounds **12a** and **12b** are presented in Figure 2. In the first step, the hydroxyl groups on the *lower rim* of calix[4]arene **1** are alkylated to yield either three (**7a**) or four (**7b**) O-propyl functionalized calix[4]arenes. In the former case, the remaining OH-group was used for conjugation with propylphthalimide (**8a**). In the next step, the *t*-butyl groups on the *upper rim* were substituted with nitro-groups, which were subsequently reduced to amines to yield compounds **10a** and **10b** (Lalor et al., 2007). The following amide coupling with tris-*t*ert-butoxycarbonylmethyl-10-carboxymethyl-1,4,7,10-tetraazacyclodocecane resulted in ligands **11a** and **11b**, which after deprotection and purification by prep-HPLC were analyzed and complexed with Tb^III^ ions. The obtained complexes **12a** and **12b** were investigated with respect to their aggregation behavior as well as their luminescent properties in a qualitative and quantitative ways.

**Figure 3:**
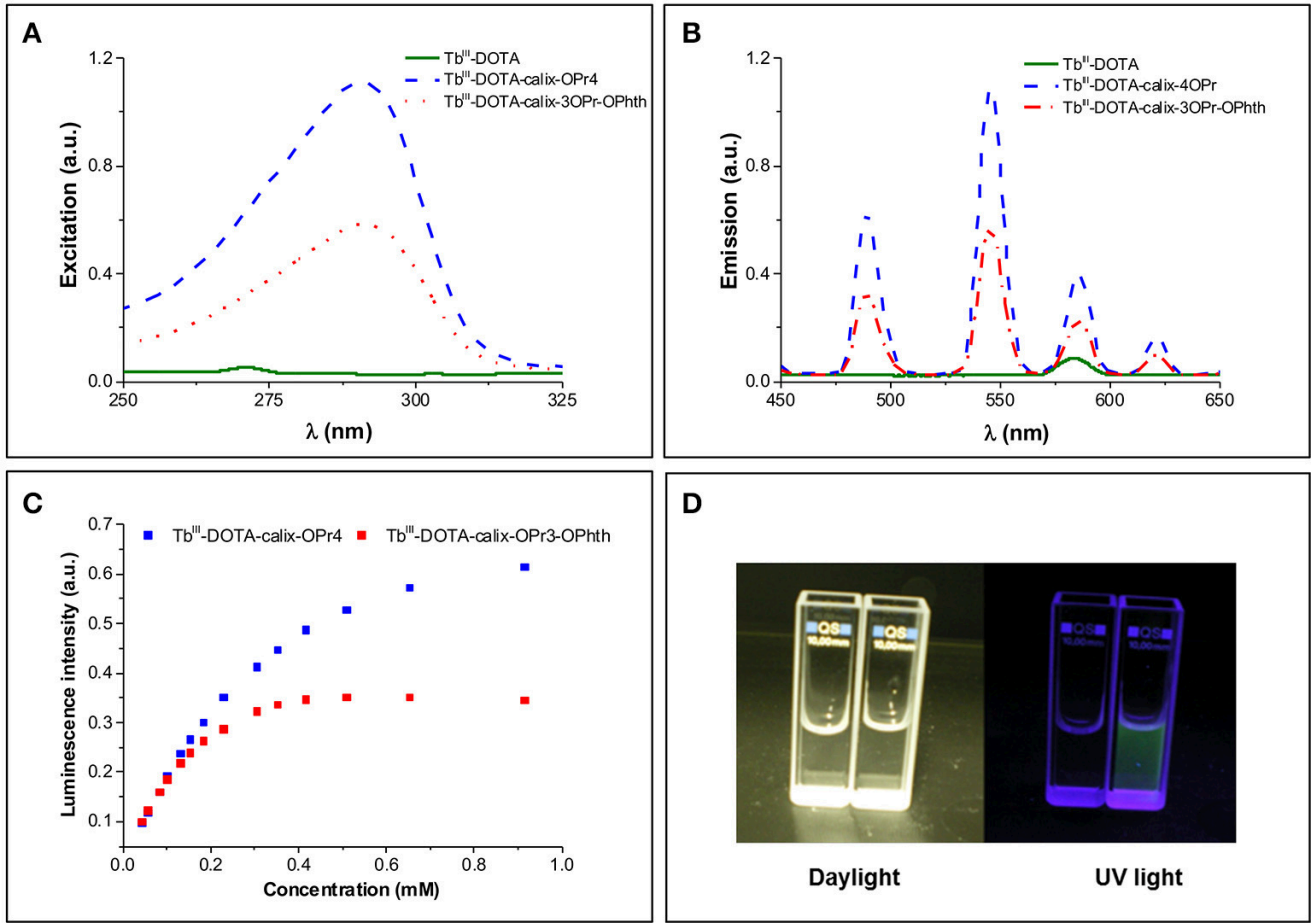
**(A)** Excitation (λ_Em_ = 545 nm) and **(B)** emission (λ_Ex_ = 290 nm) spectra of Tb^III^-DOTA, Tb-**12a** and Tb-**12b** at a Tb^III^ concentration of 3.68 mM (corresponding to a calix[4]arene concentration of 0.92 mM). The two little signals at 272 nm (excitation) and 580 nm (emission) in the Tb^III^-DOTA spectra are artifacts from light scattering (double wavelength of excitation); **(C)** The dependence of relative luminescence intensities normalized to the same initial value vs. the concentration of Tb-**12a** and Tb-**12b**; **(D)** Photographic images of Tb^III^-DOTA (left cuvette) and Tb-**12a** (right cuvette) under daylight and UV irradiation.

In order to assess the role of the calix[4]arene skeleton and of the phthalimide chromophore, the luminescence excitation and emission spectra of Tb^III^-DOTA-calix-3OPr-OPhth (**12a**) and Tb^III^-DOTA-calix-4OPr (**12b**) were recorded and compared to those of Tb^III^-DOTA. Both Tb-**12a** and Tb-**12b** under excitation at 290 nm exhibit characteristic green emission with four main bands due to ^5^${\text{D}}_{4}\to^{7}$F_*J*_ (*J* = 6-3) transitions, while the reference compound Tb^III^-DOTA under the same experimental conditions did not show detectable luminescence signal (Figures 3A,B). Upon monitoring emission at 545 nm excitation spectra of both Tb-**12a** and Tb-**12b** present broad bands in the UV range up to 315 nm. On the other hand, Tb^III^-DOTA does not exhibit pronounced transitions in this range. Therefore, in Tb-**12a** and Tb-**12b** characteristic green Tb^III^ emission could be sensitized through “antenna effect” *via* organic ligands, i.e., fully O-propyl or O-propyl and phthalimide functionalized calix[4]arene cores. Interestingly, the maximum luminescence intensity of the two calix[4]arene-complexes shows a dramatically different variation with increasing concentration of the solutions. At concentrations up to ~0.1 mM, there is no difference in their luminescence intensities, which remain equally strong for both compounds with a linear correlation to the concentration (Figures 3C,D). Upon further increase of the concentration, the luminescence enhancement becomes non-linear. At 1 mM concentration, the emission intensity is two times higher for Tb-**12b** compared to Tb-**12a**. While the luminescence intensity of Tb-**12b** is still increasing above this concentration, that of Tb-**12a** goes through a maximum at 0.5 mM and then slightly decreases. Likewise, a maximum luminescence intensity is found for Tb-**12b** above the concentration of 5 mM (Supplementary Figure 3).

To further quantify the photophysical properties, the excitation and emission spectra of Tb-complexes of calix[4]arenes **12a** and **12b** were acquired in the solid state and at different concentrations in solution (Supplementary Figures 4, 5). It should be noted that in the emission spectra of **12a** and **12b** the crystal-field splitting of Tb^III^ ^5^${\text{D}}_{4}\to^{7}$F_*J*_ (*J* = 6-3) transitions is very similar for the samples in the solid state and solutions of different concentrations reflecting indirectly that the coordination environment around the Tb^III^ ions remains the same upon such variations. The absolute quantum yields upon ligands excitation ($Q_{Tb}^{L}$) and observed luminescence lifetimes (τ_obs_) upon monitoring Tb^III^ emission at 545 nm were also determined (Table 1). When comparing the values of τ_obs_ and $Q_{Tb}^{L}$ for Tb-**12a** and Tb-**12b** in solution, the presence of the phthalimide chromophore in the molecule has essentially no effect on these parameters. This can be likely rationalized by the fact that the distance between the phthalimide and the Tb^III^ center is too long (>15 Å) for an efficient energy transfer (Vázquez López et al., 2010). In contrast, in amorphous solid state the orientation of the molecules is random and the phthalimide units can be located closer to the Tb^III^-DOTA moieties, making non-covalent energy transfer and Tb^III^ sensitization through this way possible (Howell et al., 2003). Quantum yield values of Tb-**12a** and Tb-**12b** in the solid state are higher by 7.5 and 2.7 times, respectively, compared to these in 0.2 mM solutions, while lifetimes are lower by 25–30%. Such behavior points that in the solid state non-radiative processes are minimized and/or sensitization efficiencies are improved. Higher increase of the $Q_{Tb}^{L}$-value in the case of Tb-**12a** compared to the Tb-**12b** might be caused by an appearance of an additional sensitization pathway. Indeed, if we assume that the main energy transfer mechanism is of Förster type (dipole-dipole), the sensitization efficiency depends significantly on distance and is proportional to (1/*r*_Tb−L_)^−6^. Thus, small changes in the distance between the chromophore and Tb^III^ ion may have significant effects on the sensitization efficiency. This is also reflected in 3.1-times higher quantum yield value for Tb-**12a** compared to Tb-**12b** while observed luminescence lifetimes are the same for both complexes (Table 1). Moreover, slight broadening and red-shifting of the excitation band is observed for Tb-**12a** vs. Tb-**12b** is observed for Tb-**12a** vs. Tb-**12b** that can indicate that other lower-energy levels are involved in the sensitization of the former complex (Supplementary Figure 4, left). Since this extra shoulder, only present in the solid-state excitation spectrum, overlaps with one of the UV absorption peaks of the phthalimide moiety (Supplementary Figure 6), it may indicate that in the solid state the phthalimide group can indeed participate in the sensitisation of the Tb^III^ luminescence. In addition, the quantum yield values confirm the trend that was observed qualitatively for solutions with different concentrations (Figure 3C vs. Table 1): they increase upon dilution evidencing a non-linear correlation between the luminescence intensity and the concentration of the investigated compounds. Concentration of solutions has also effect on luminescence lifetimes. It should be noted here that for all solutions luminescence decay curves could be best fitted by mono-exponential functions reflecting the presence of only one type of emissive Tb^III^-containing species. The values of τ_obs_ increase by 1.8–2.0 times when going from 2 to 0.2 mM solutions, i.e., following the same trend as absolute quantum yield values.

**Table 1:** Photo-physical parameters of Tb^III^ complexes in the solid state and aqueous solutions at 298 K.^a^

**Compound**	**State/Solvent**	***c* (mM)**	**τ_obs_ (ms)**	**$Q_{Tb}^{L}$ (%)**
Tb-**12a**	Solid	–	1.2(1)^b^	5.87(9)
	H_2_O	0.04	1.52(1)	0.78(7)
		0.2	1.51(1)	0.65(1)
		2	0.83(5)	0.51(2)
Tb-**12b**	Solid	–	1.2(1)^b^	1.89(3)
	H_2_O	0.2	1.6(1)	0.71(3)
		2	0.79(5)	0.38(6)

a *Under excitation at 300 nm. Standard deviations (2σ) are given between parentheses; Estimated relative errors: τ_obs_, ±5%; $Q_{Tb}^{L}$, ±10%*.

b *The longest values are presented. Luminescence decay curves were best fitted by biexponential functions however the impact of the second short-lived lifetime value (0.17–0.22 ms) was only 5–6%*.

To get a hint about possible reasons of such changes of luminescent parameters, we have investigated the aggregation of the complexes in details. It has been previously described that the DOTA-functionalized calix[4]arene (Gd-**12b**) is highly amphiphilic and tends to aggregate in aqueous solution. At concentrations above 0.21 mM, they form micelles with a hydrodynamic radius of 2.2 nm and a narrow size distribution (Schühle et al., 2009). This critical micelle concentration (*cmc*) was determined for the Gd^III^ complex by measuring the water proton relaxation rates as a function of the concentration.

**Figure 4:**
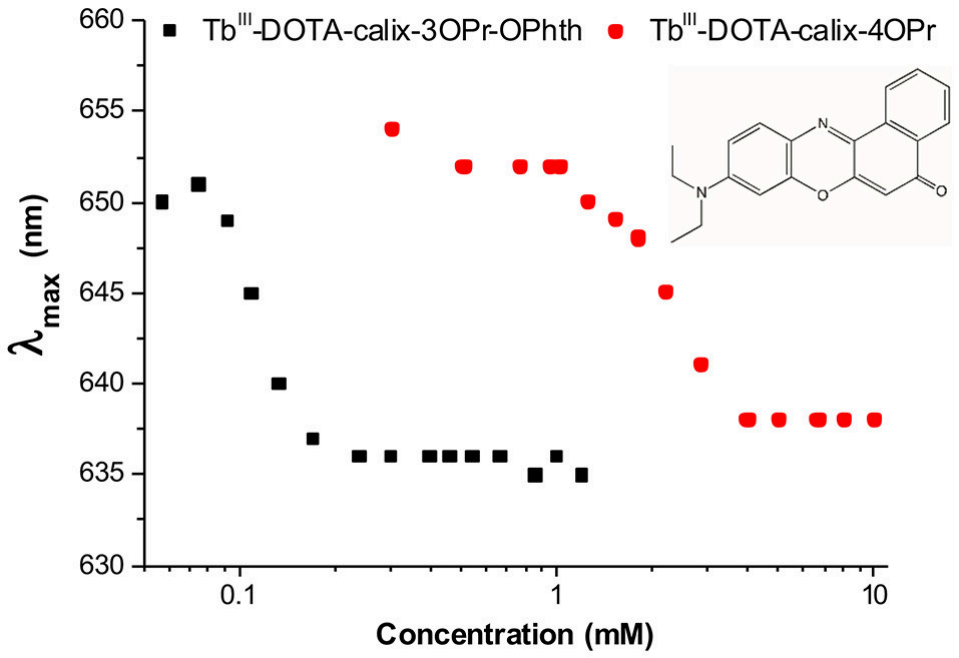
Wavelength of the fluorescence emission maximum (λ_max_) vs. Tb^III^ concentration in solutions of Tb^III^-complexes **12a** (black) and **12b** (red) in the presence of Nile red (λ_Ex_ = 550 nm).

In the current study, the aggregation was demonstrated by using Nile red, a dye exhibiting fluorescence changes in the emission maximum upon changes in the chemical environment. In the samples containing Nile red and increasing concentrations of the Tb^III^ complexes **12a** and **12b**, the fluorescence emissions undergoes strong blue-shifts and intensity increase, which can be associated with a decreasing hydrophilicity and polarity of the environment (Figure 4). Indeed, as the amphiphilic calix[4]arene complexes aggregate to micelles, the Nile red will enter the internal micellar core and will experience a more hydrophobic environment. From this experiment, the critical micelle concentrations of Tb^III^-DOTA-calix-3OPr-OPhth (Tb-**12a**) and Tb^III^-DOTA-calix-4OPr (Tb-**12b**) were determined to be 0.12 and 2.3 mM, respectively (Supplementary Figure 7). Obviously, the presence of phthalimide dramatically increases the hydrophobicity of the *lower rim* forces the molecules to aggregate at lower concentrations and thus leads to a lower *cmc*. Interestingly, the determined *cmc*-values correlate nicely with the maxima of the concentration dependent luminescence intensities (Figure 3C and Supplementary Figure 3), which are around 0.35 and 1.6 mM for Tb-**12a** and Tb-**12b**, respectively. This evidences that aggregation of the calix[4]arenes leads to a significant decrease in the intensity of Tb^III^ luminescence. As the aromatic systems come closer to each other, non-radiative energy transfer from the excited π-systems becomes more likely which reduces luminescence quantum yields of the aggregated complexes. As a result, decreased quantum yields and shorter luminescence lifetimes are observed.

**Figure 5:**
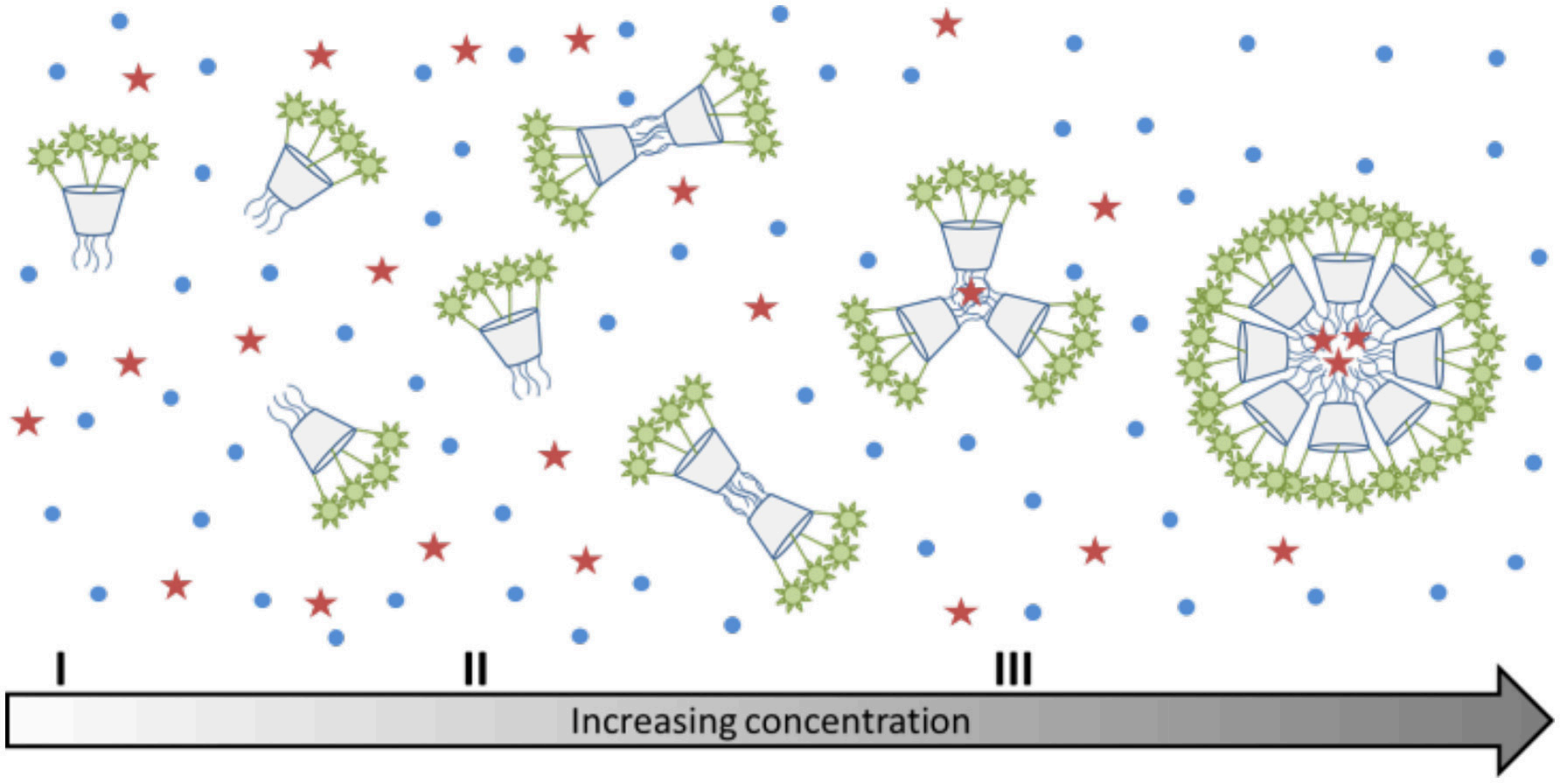
**(I–III)** Schematic representation of the aggregation behavior of calix[4]arenes **7a** and **7b** with increasing concentration. The red stars indicate the location of Nile red, which can only enter the micelles, when there is a sufficiently large hydrophobic space.

Surprisingly, the *cmc*-value previously determined by relaxometric measurements for the Gd-**12b** analog (0.21 mM) is significantly lower than *cmc* found in the Nile red experiment for Tb-**12b** (2.3 mM). It is very unlikely that this is due to the different ions complexed in the DOTA chelates, as lanthanides in general have very similar chemical properties. Furthermore, Gd and Tb are neighbors in the lanthanide series thus have similar ionic radii (difference ~0.1 Å) and identical charge. The almost one order of magnitude difference in *cmc* can rather be explained by the different experimental methods applied for its determination. The relaxometric method used in the case of the paramagnetic analog Gd^III^-**12b** is based on the effect of the rotational motion on the *r*_1_ relaxivity (longitudinal relaxation rate expressed in mM^−1^s^−1^ of Gd^III^). Indeed, at medium fields, the relaxivity increases with the increasing rotational correlation time, τ_R_ (slower motion) of the complex. While τ_R_ already changes when as few as two molecules start interacting (Figures 5I,II), the creation of noticeable hydrophobic compartments in micelles leading to an observable change in the fluorescence of the dye only starts at a higher degree of aggregation, when several amphiphilic molecules aggregate (Figure 5III). Therefore, the *cmc* determined by the fluorescence method using Nile red is significantly higher than the one obtained *via* relaxivity measurements. Previous studies have already pointed out that the *cmc*-value obtained can be dependent on the nature of the physical parameter monitored to assess the aggregation and that premicellar aggregation is often responsible for anomalies of various physical parameters (Pérez-Rodríguez et al., 1999; He et al., 2004).

It is interesting to note that the concentration dependent luminescence intensities of Tb-**12b** (Figure 3C) start to show a deviation from linearity at ~0.25 mM concentration, which corresponds to the *cmc* determined from the relaxivity method for the Gd^III^ analog. These results nicely demonstrate that micelle formation is not a sharp transition, but it expands over a concentration range that might cover more than an order of magnitude. As the various techniques used to determine the critical micelle concentration sense different physical parameters, they might lead to method-dependent *cmc*-values.

Upon aggregation of the functionalized calix[4]arenes, the hydrophilic Tb^III^-DOTA moieties on the *upper rim* will always face the aqueous phase. The lifetimes observed for the aggregated state (0.83 and 0.79 ms) are smaller than those measured for the monomers (1.51 and 1.60 ms) of **12a** and **12b**, respectively. This points to a better protection of the Tb^III^ ion from non-radiative deactivations in the case of the monomers. An increase of Tb-luminescence lifetimes occurs upon exclusion of water molecules from the first coordination sphere of the metal ion (Murru et al., 1993; Chen et al., 2013). In our case, a possible explanation of the shortening of luminescence lifetimes found for the micellar Tb^III^-DOTA-functionalized calix[4]arenes could be an enhanced cross-relaxation between Tb^III^ ions and the formation of a more defined second coordination sphere around the micelle consisting of water molecules clustered *via* hydrogen bonds.

## Conclusions

In summary, we have synthesized the first luminescent lanthanide complex based on a calix[4]arene platform with sufficient stability due to the presence of four DOTA chelating units for potential biological use. We have evidenced for this prototype Tb^III^ probe that the calix[4]arene backbone is actively participating to the excitation process of the Tb ^III^-centered luminescence, while the chromophore introduced on the rim opposite to the lanthanide chelate is localized too far to provide efficient sensitization. The substitution pattern of the *lower rim* dictates the aggregation phenomena observed in aqueous solution and can be exploited to tailor the physical-chemical properties of the compound. This micellar aggregation of the calix[4]arene derivatives has a strong impact on their luminescence properties. Upon increasing the complex concentration, the luminescence intensities linearly increase up to the range where micellar aggregation starts to occur, and then they decline. Taking into account these peculiar properties, the future perspectives of the described systems may be based on the complexation of both luminescent Ln^III^ and paramagnetic Gd^III^ ions within the same molecule. This in turn, could yield dual MR/optical imaging probes in a straightforward manner rendering the calix[4]arene backbone a valuable building block for the design of imaging probes.

## Author contributions

All authors have contributed equally to the experimental as well as to the writing process of the manuscript. All authors read and approved the final version of the manuscript.

### Conflict of interest statement

The authors declare that the research was conducted in the absence of any commercial or financial relationships that could be construed as a potential conflict of interest.

## References

[B1] Baldini L. Cacciapaglia R. Casnati A. Mandolini L. Salvio R. Sansone F.. (2012). Upper rim guanidinocalix[4]arenes as artificial phosphodiesterases. J. Org. Chem. 77, 3381–3389. 10.1021/jo300193y 22364173

[B2] Blanchard S. Le Clainche L. Rager M.-N. Chansou B. Tuchagues J.-P. Duprat A. F. (1998). Calixarene-based copper(I) complexes as models for monocopper sites in enzymes. Angew. Chem. Int. Ed. 37, 2732–2735. 10.1002/(SICI)1521-3773(19981016)37:19<2732::AID-ANIE2732>3.0.CO;2-7 29711612

[B3] Bonnet C. S. Pellegatti L. Buron F. Shade C. M. Villette S. Kubicek V.. (2010). Hydrophobic chromophore cargo in micellar structures: a different strategy to sensitize lanthanide cations. Chem. Commun. 46, 124–126. 10.1039/B918881A 20024314

[B4] Bunzli J. C. G. Froidevaux P. Harrowfield J. M. (1993). Complexes of lanthanoid salts with macrocyclic ligands. 41. Photophysical properties of lanthanide dinuclear complexes with p-tert-butylcalix[8]arene. Inorg. Chem. 32, 3306–3311. 10.1021/ic00067a019

[B5] Bünzli J.-C. G. (2015). On the design of highly luminescent lanthanide complexes. Coord. Chem. Rev. 293–294, 19–47. 10.1016/j.ccr.2014.10.013

[B6] Bunzli J.-C. G. Ihringer F. Dumy P. Sager C. Rogers R. D. (1998). Structural and dynamic properties of calixarene bimetallic complexes: solution versus solid-state structure of dinuclear complexes of EuIII and LuIII with substituted calix[8]arenes. J. Chem. Soc. Dalton Trans. 497–504. 10.1039/a706933b

[B7] Chen G. Wardle N. J. Sarris J. Chatterton N. P. Bligh S. W. A. (2013). Sensitized terbium(iii) macrocyclic-phthalimide complexes as luminescent pH switches. Dalton Trans. 42, 14115–14124. 10.1039/c3dt51236c 23938770

[B8] Darwent J. R. Dong W. Flint C. D. Sharpe N. W. (1993). Intermolecular energy transfer between phenanthrene and lanthanide ions in aqueous micellar solution. J. Chem. Soc. Faraday Trans. 89, 873–880. 10.1039/ft9938900873

[B9] Escabi-Perez J. R. Nome F. Fendler J. H. (1977). Energy transfer in micellar systems. steady state and time resolved luminescence of aqueous micelle solubilized naphthalene and terbium chloride. J. Am. Chem. Soc. 99, 7749–7754. 10.1021/ja00466a001

[B10] Fischer C. Sarti G. Casnati A. Carrettoni B. Manet I. Schuurman R.. (2000). 2,2′-Bipyridine lariat calixcrowns: a new class of encapsulating ligands forming highly luminescent Eu3+ and Tb3+ complexes. Chem. Eur. J. 6, 1026–1034. 10.1002/(SICI)1521-3765(20000317)6:6<1026::AID-CHEM1026>3.0.CO;2-C 10785823

[B11] Gutsche C. D. Lin L.-G. (1986). Calixarenes 12. Tetrahedron 42, 1633–1640. 10.1016/S0040-4020(01)87580-3

[B12] Gutsche C. D. Muthukrishnan R. (1978). Calixarenes. 1. Analysis of the product mixtures produced by the base-catalyzed condensation of formaldehyde with para-substituted phenols. J. Org. Chem. 43, 4905–4906. 10.1021/jo00419a052

[B13] Gutsche C. D. Iqbal M. (1990). p-tert-Butylcalix[4]arene. Org. Synth. 68, 234–237. 10.15227/orgsyn.068.0234

[B14] He X. Liang H. Huang L. Pan C. (2004). Complex microstructures of amphiphilic diblock copolymer in dilute solution. J. Phys. Chem. B 108, 1731–1735. 10.1021/jp0359337

[B15] Howell C. R. Edwards H. S. Gajadhar-Plummer S. A. Kahwa A. I. Mcpherson L. G. Mague T. J. (2003). Phthalimides: supramolecular interactions in crystals, hypersensitive solution 1H-NMR dynamics and energy transfer to Europium(III) and Terbium(III) States. Molecules 8, 565–592. 10.3390/molecules8070565

[B16] Kelderman E. Verboom W. Engbersen J. F. J. Reinhoudt D. N. Heesink G. J. T. Van Hulst N. F. (1992). Nitrocalix [4]arenes as molecules for second-order nonlinear optics. Angew. Chem. Int. Ed. Engl. 31, 1075–1077. 10.1002/anie.199210751

[B17] Klimentová J. Vojtíšek P. (2007). New receptors for anions in water: Synthesis, characterization, X-ray structures of new derivatives of 5,11,17,23-tetraamino-25,26,27,28-tetrapropyloxycalix[4]arene. J. Mol. Struct. 826, 48–63. 10.1016/j.molstruc.2006.04.016

[B18] Konczyk J. Nowik-Zajac A. Kozlowski C. A. (2016). Calixarene-based extractants for heavy metal ions removal from aqueous solutions. Sep. Sci. Technol. 51, 2394–2410. 10.1080/01496395.2016.1209219

[B19] Lalor R. Digesso J. L. Mueller A. Matthews S. E. (2007). Efficient gene transfection with functionalised multicalixarenes. Chem. Commun. 4907–4909. 10.1039/b712100h 18361365

[B20] Lawal O. Iqbal K. S. J. Mohamadi A. Razavi P. Dodd H. T. Allen M. C. (2009). An artificial sodium ion channel from calix[4]arene in the 1,3-alternate conformation. Supramol. Chem. 21, 55–60. 10.1080/10610270802528307

[B21] Modi K. Panchal U. Mehta V. Panchal M. Kongor A. Jain V. K. (2016). Propyl phthalimide-modified thiacalixphenyl[4]arene as a “turn on” chemosensor for Hg(II) ions. J. Lumin. 179, 378–383. 10.1016/j.jlumin.2016.07.019

[B22] Murru M. Parker D. Williams G. Beeby A. (1993). Luminescence behaviour of stable europium and terbium complexes of tetraaza phosphinates: efficient through-space energy transfer from phenyl to terbium. J. Chem. Soc. Chem. Commun. 1116–1118. 10.1039/c39930001116

[B23] Pérez-Rodríguez M. Varela L. M. García M. Mosquera V. Sarmiento F. (1999). Conductivity and relative permittivity of sodium n-dodecyl sulfate and n-dodecyl trimethylammonium bromide. J. Chem. Eng. Data 44, 944–947. 10.1021/je980301c

[B24] Sato N. Yoshida I. Shinkai S. (1993). Energy-transfer luminescence of lanthanide ions complexed with water-soluble calix[n]arenes. Chem. Lett. 22, 1261–1264. 10.1246/cl.1993.1261

[B25] Schühle D. T. Peters J. A. Schatz J. (2011). Metal binding calixarenes with potential biomimetic and biomedical applications. Coord. Chem. Rev. 255, 2727–2745. 10.1016/j.ccr.2011.04.005

[B26] Schühle D. T. Polasek M. Lukes I. Chauvin T. Toth E. Schatz J. (2010). Densely packed Gd(iii)-chelates with fast water exchange on a calix[4]arene scaffold: a potential MRI contrast agent. Dalton Trans. 39, 185–191. 10.1039/B917673J 20023949

[B27] Schühle D. T. Schatz J. Laurent S. Vander Elst L. Muller R. N. Stuart M. C. A.. (2009). Calix[4]arenes as molecular platforms for magnetic resonance imaging (MRI) contrast agents. Chem. Eur. J. 15, 3290–3296. 10.1002/chem.200802099 19206118

[B28] Sreenivasu Mummidivarapu V. V. Hinge V. K. Rao C. P. (2015). Interaction of a dinuclear fluorescent Cd(II) complex of calix[4]arene conjugate with phosphates and its applicability in cell imaging. Dalton Trans. 44, 1130–1141. 10.1039/C4DT01726A 25412362

[B29] Vázquez López M. Eliseeva S. V. Blanco J. M. Rama G. Bermejo M. R. Vázquez M. E. (2010). Synthesis and photophysical properties of LnIII–DOTA–Bipy complexes and LnIII–DOTA–Bipy–RuII coordination conjugates. Eur. J. Inorg. Chem. 2010, 4532–4545. 10.1002/ejic.20100051

