# Supplementary material for: Luminescence Properties of Self-Aggregating TbIII-DOTA-Functionalized Calix[4]arenes
Source: Front Chem. 2018 Jan 30;6:1. doi: 10.3389/fchem.2018.00001 (PMC5797610; doi:10.3389/fchem.2018.00001)
Supplement: Supplementary file 1 [file DataSheet1.PDF]

Supplementary Material

Luminescence Properties of Self-Aggregating Tb^{III}-DOTA-Functionalized Calix[4]arenes

Florian Mayer[1], Sriram Tiruvadi Krishnan[1], Daniel T. Schühle[1], Svetlana V. Eliseeva[2], Stéphane Petoud[2], Éva Tóth[2], and Kristina Djanashvili[1,2,3]*

***Correspondence:** k.djanashvili@tudelft.nl

[1] Department of Biotechnology, Delft University of Technology, Delft, The Netherlands

[2] Centre de Biophysique Moléculaire, UPR 4301 CNRS, Université d'Orléans, Orléans, France

[3] Le Studium, Loire Valley Institute for Advanced Studies, 1 Rue Dupanloup, 45000 Orléans, France

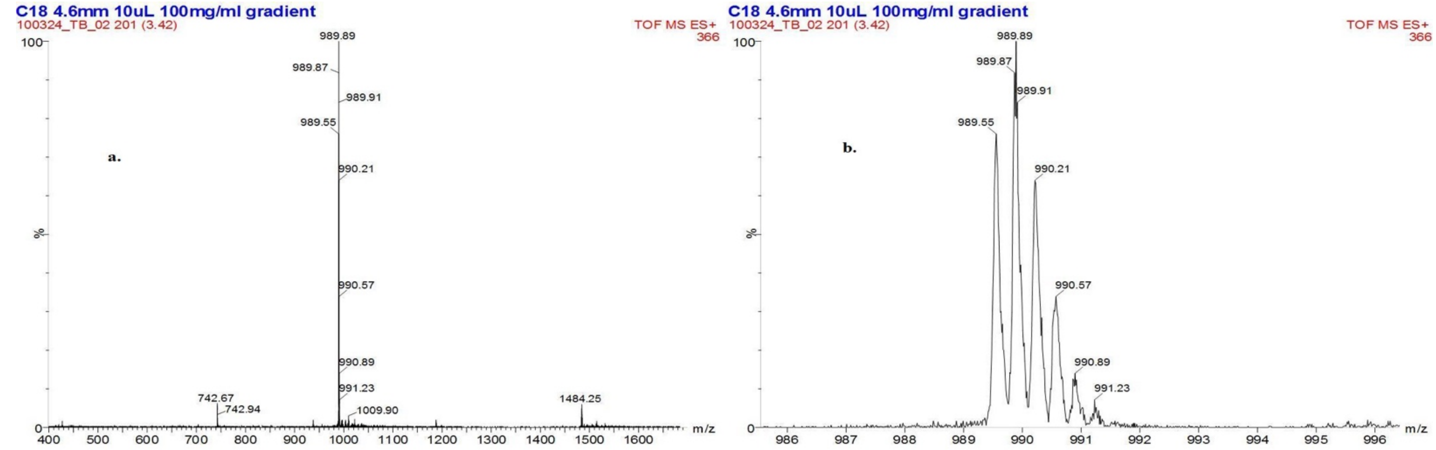

Supplementary Figure 1. ESI-HRMS of Tb-**12a:** a) $[M+4H]^{4+}$ (742.67), $[M+3H]^{3+}$ (989.89), $[M+2H]^{2+}$ (1484.25); b) $[M+3H]^{3+}$ peak zoomed in to show the isotopic pattern of Tb^{III} ions.

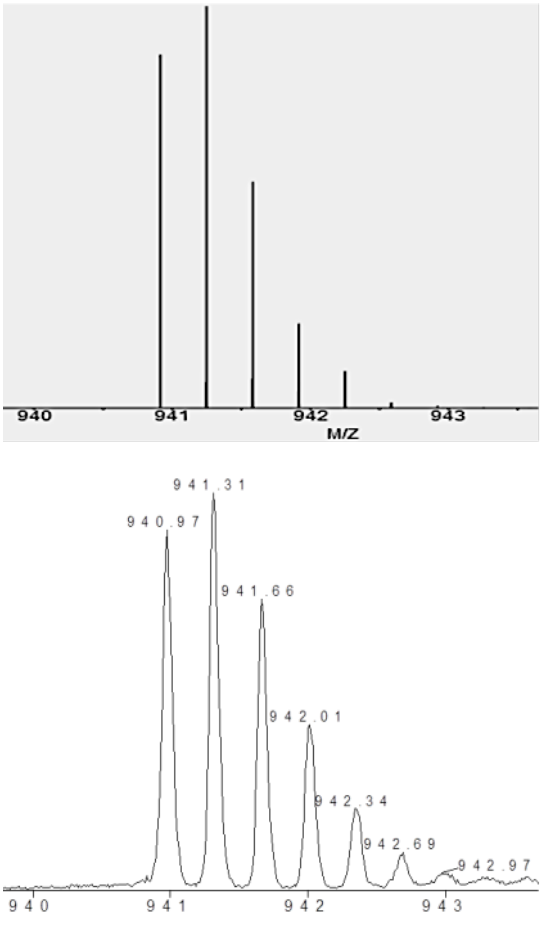

Supplementary Figure 2. ESI-HRMS of Tb-**12b** $(M+3H)^{3+}$

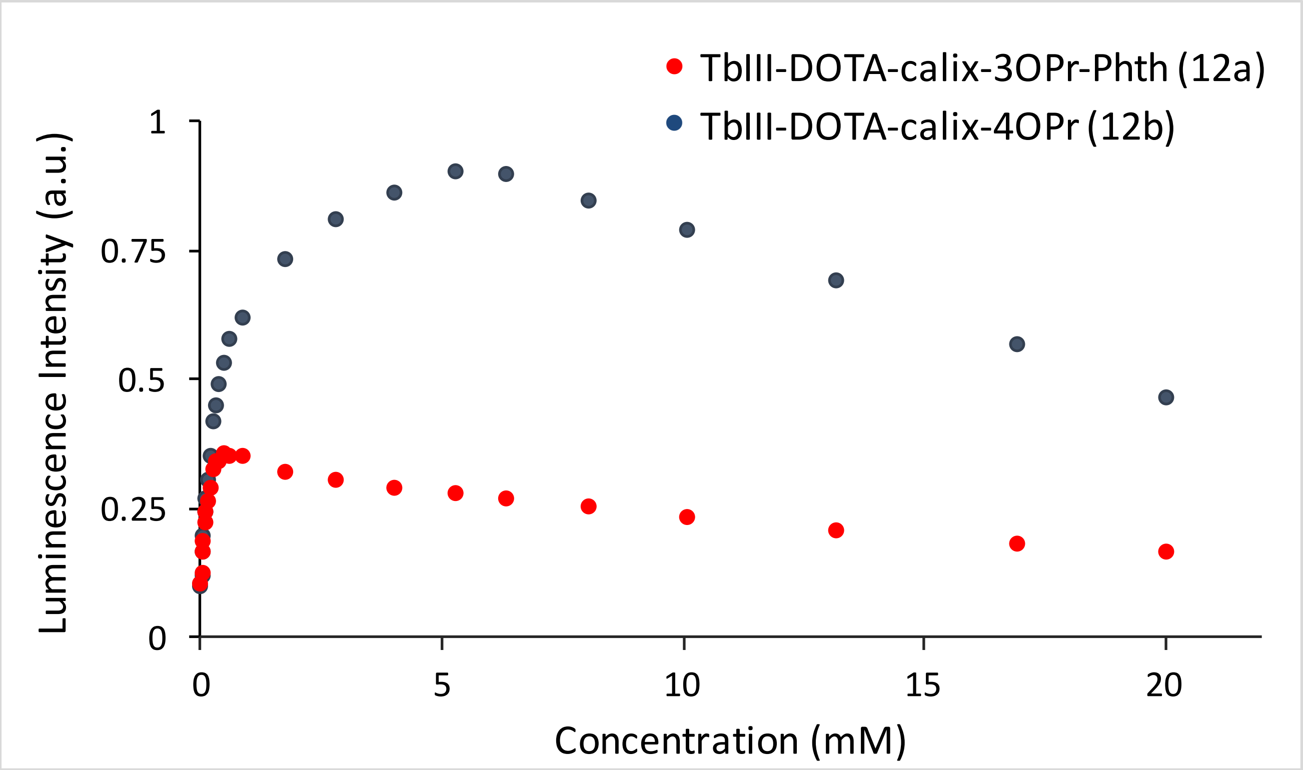

Supplementary Figure 3. Luminescence intensity of Tb^{III}-DOTA-calix-3OPr-Phth **(12a)** and Tb^{III}-DOTA-calix-4OPr **(12b)** at 545 nm vs. concentration.

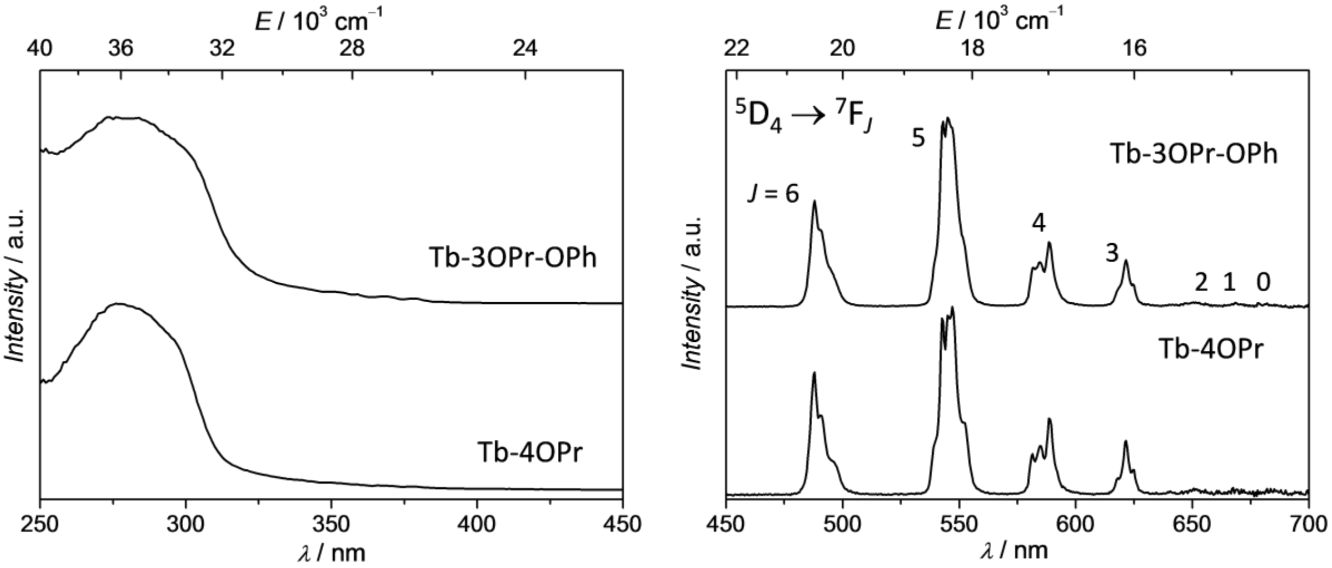

Supplementary Figure 4. Excitation (left, λ_{em} = 545 nm) and emission (right, λ_{ex} = 300 nm) spectra of Tb^{III} complexes in solid state at room temperature.

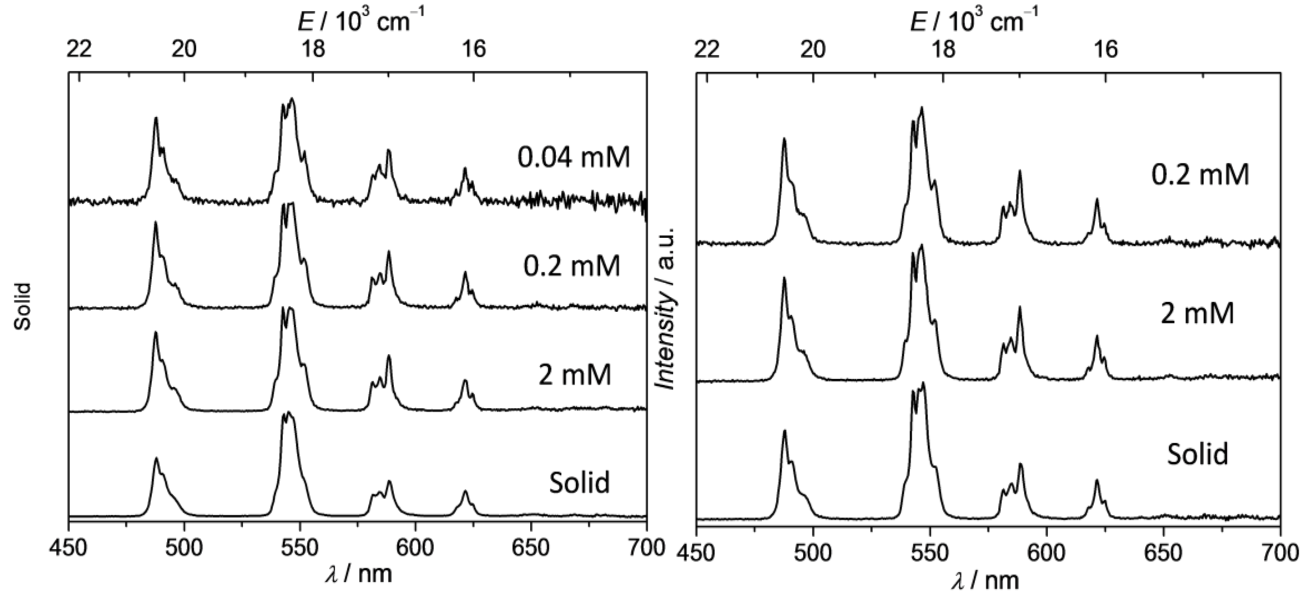

Supplementary Figure 5. Comparison of emission spectra (λ_{ex} = 300 nm) of complexes Tb^{III}-DOTA-calix-3OPr-Phth **(12a**, left) and Tb^{III}-DOTA-calix-4OPr **(12b**, right) at room temperature.

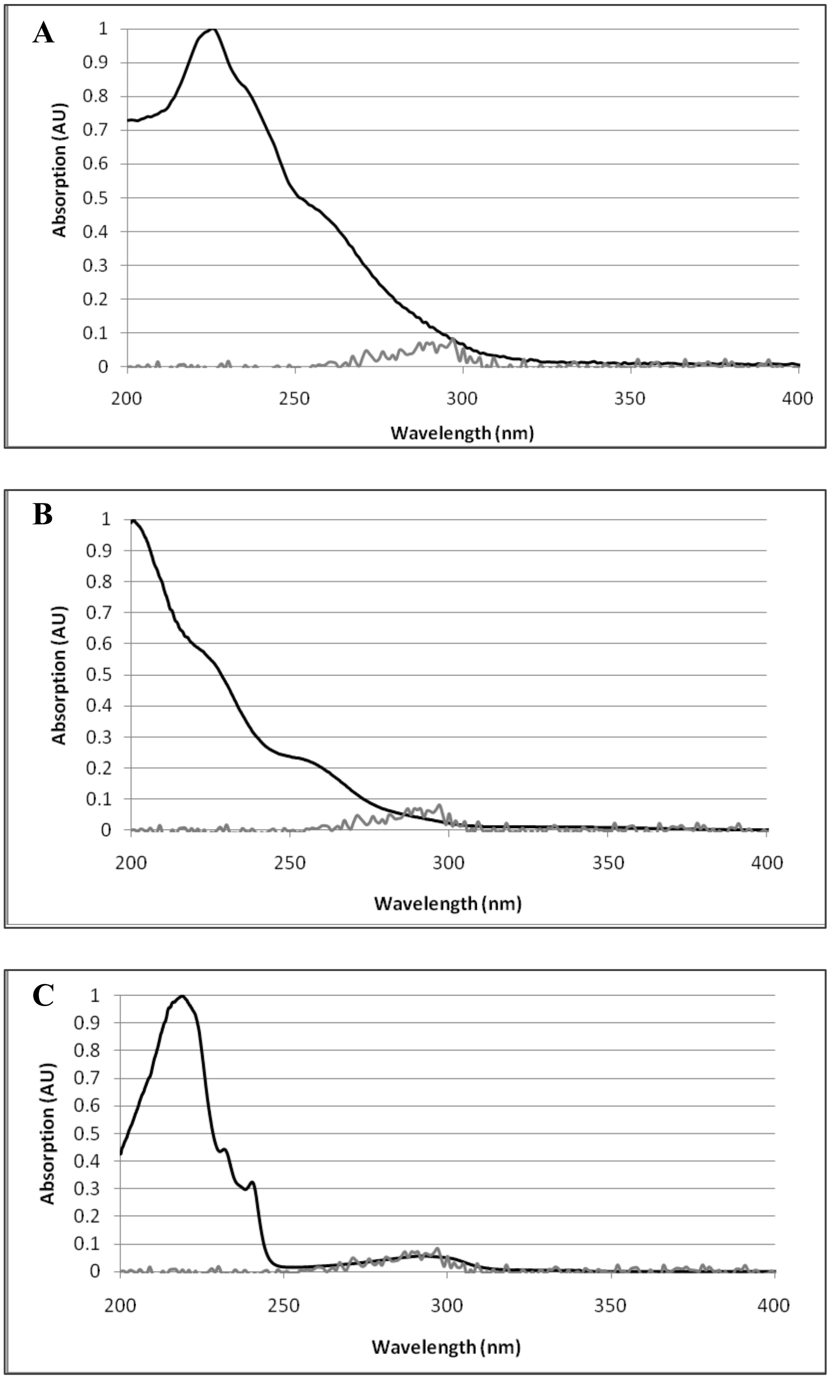

Supplementary Figure 6. UV absorption spectra of **Tb-12a** (A), **Tb-12b** (B) and N-(3-bromopropyl) phthalimide (C) and the excitation spectra of **12a** at 540 nm (λ_{max} of Tb^{III}, [G.S.R. Raju et al., *Spectro. Lett.* **2006**, *39*, 487]).

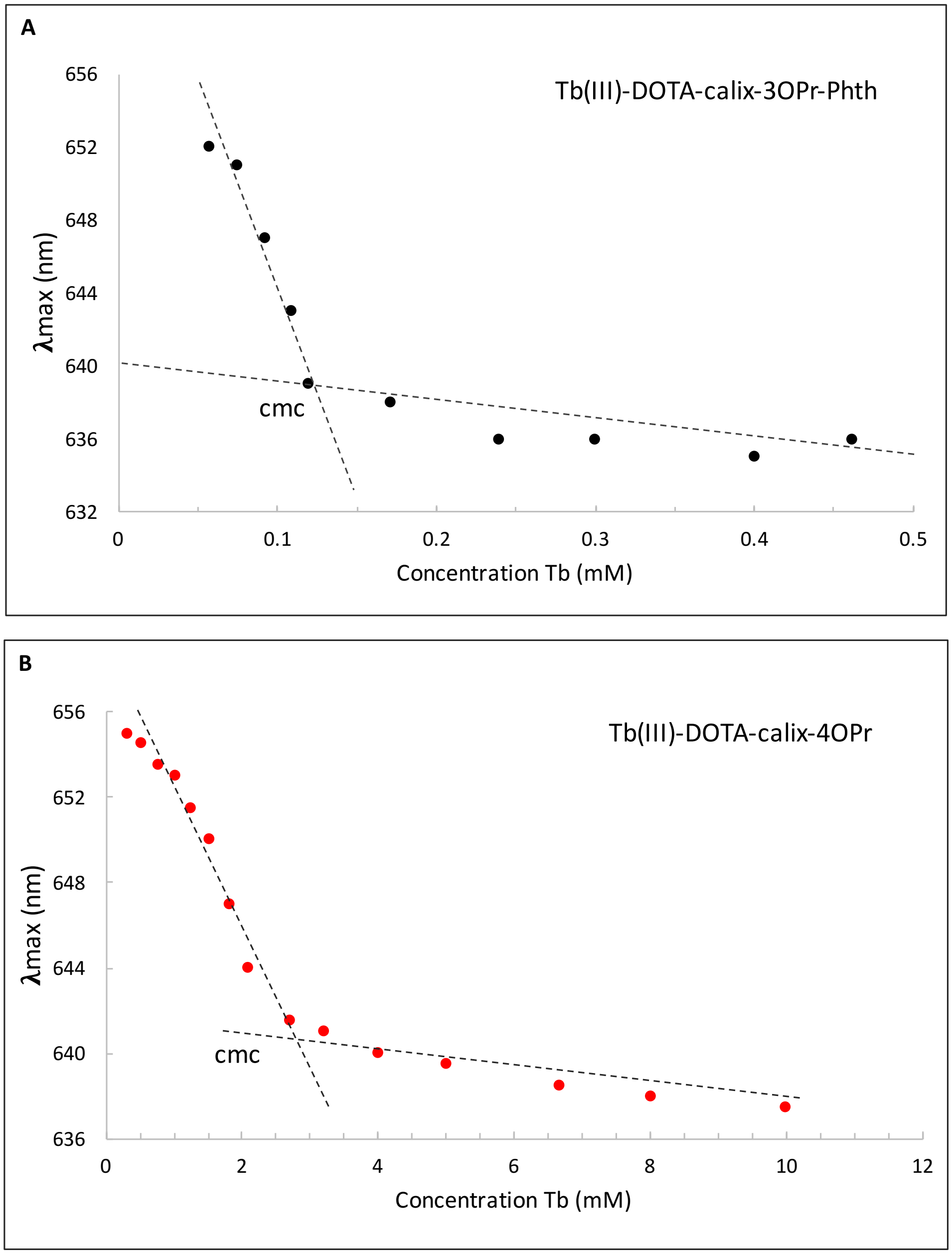

Supplementary Figure 7. Determination of the *cmc* values of Tb^{III}-complexes **12a** (A) and **12b** (B) in the presence of Nile red (λ_{Ex} = 550 nm) by measuring wavelength of the fluorescence emission maximum (λ_{max}).
